# Glycerol Monolaurate Alleviates Oxidative Stress and Intestinal Flora Imbalance Caused by Salinity Changes for Juvenile Grouper

**DOI:** 10.3390/metabo12121268

**Published:** 2022-12-15

**Authors:** Xuehe Li, Dongwenjun Zhu, Minling Mao, Jianwei Wu, Qihui Yang, Beiping Tan, Shuyan Chi

**Affiliations:** 1 College of Fisheries, Guangdong Ocean University, Zhanjiang 524088, China; 2 Aquatic Animals Precision Nutrition and High Efficiency Feed Engineering Research Center of Guangdong Province, Zhanjiang 524088, China; 3 Guangdong Provincial Key Laboratory of Aquatic Animal Disease Control and Healthy Culture, Zhanjiang 524088, China

**Keywords:** glycerol monolaurate, hybrid grouper, salinity change, oxidative stress, antioxidant capacity, intestinal flora

## Abstract

Groupers with an initial body weight of 9.10 ± 0.03 g were selected to investigate whether dietary addition of 0 (G0) and 1800 mg/kg glycerol monolaurate (GML, G1800) could alleviate the oxidative stress response and intestinal flora imbalance after 0, 6, 12, and 24 h of salinity change in grouper. Experimental results show that the dietary addition of GML significantly reduced the liver MDA content and increased the SOD activity of grouper. The gene expression of CAT and SOD increased and then decreased with time after adding 1800 mg/kg GML, and the highest values were significantly higher than those of the control group. Salinity change had a slight effect on the top four intestinal flora composition of grouper at 0, 12, and 24 h, with changes occurring only at 6 h when Cyanobacteria replaced Actinobacteria. The addition of dietary GML slowed down the intestinal flora disorder, inhibited the colonization of harmful bacterium *Vibrio*, and promoted the abundance of beneficial bacterium *Bacillus*. In conclusion, dietary GML significantly reduced the oxidative damage caused by sudden changes in salinity, improved the antioxidant capacity, and alleviated the intestinal flora imbalance in juvenile grouper.

## 1. Introduction

With the development of intensive aquaculture, aquatic animals are subjected to increasing stress factors, such as excessive density, water pollution, shock, and sudden changes in salinity and temperature. Environmental stresses may induce excessive production or accumulation of reactive oxygen radicals (ROS) in aquatic animals. They could affect the antioxidant protection capacity of aquatic animals, thus causing oxidative stress in fish and even leading to various disease outbreaks [1,2,3].

Previous studies have shown that a variety of essential nutrients (e.g., proteins, amino acids, fatty acids, carbohydrates, and vitamins) and additives (e.g., enzymes and immunopolysaccharides) could modulate the immune system of aquatic animals and alleviate the stress experienced by them in the environment. This modulation is one of the effective means of enhancing stress resistance, and it could be a stress management strategy to improve the overall performance of aquatic animals [4,5,6]. Functional feed additives, as one of the antistress strategies, could play an indirect role in promoting growth by improving immune function, reducing oxidative stress, and enhancing disease resistance rather than directly providing additional nutrients necessary for growth [7].

Glycerol monolaurate (GML) is a natural compound composed of glycerol and lauric acid, a derivative of fatty acids, with remarkable antibacterial, antiviral, and anti-inflammatory properties [8]. Although the beneficial effects of GML are gradually being recognized, most of them are limited to livestock and poultry animals. The applications of GML in aquaculture is relatively few, and the studies have been mainly focused on *Danio rerio* [9], *Larimichthys crocea* [10], *Lateolabrax maculatus* [11], *Litopenaeus vannamei* [12], and *Pelodiscus sinensis* [13]. The above experimental results show that GML can significantly improve growth performance, digestive capacity, immune index, antioxidant activity, muscle quality, and lipid metabolism. Moreover, studies on whether GML supplementation could reduce oxidative stress and decrease changes in intestinal flora in aquatic organisms are not sufficient.

Hybrid grouper (*Epinephelus fuscoguttatus*♀ × *Epinephelus lanceolatus*♂) has become one of the most important breeding species along the southeast coast of China because of its fast growth rate, high nutritional value, and disease resistance [14]. The annual production of grouper reached 204,200 tons in 2021 [15]. In recent years, the continuous expansion of grouper culture scale and increasing culture density, coupled with the influence of water environment changes and other factors, has caused oxidative stress of grouper and frequent occurrence of various diseases, bringing serious economic losses to its culture industry [1,16]. The hybrid grouper was chosen as the subject of this study, which aimed to investigate whether adding GML to the diet could improve the antioxidant capacity of grouper, mitigate oxidative stress, and protect intestinal health.

## 2. Materials and Methods

### 2.1. Diets Preparation

Two experimental diets were prepared by adding 1800 mg/kg GML (G1800) to the basal diet (G0) (Table 1). Firstly, all powdered ingredients were passed through a 60-mesh screen and mixed using a uniform V-mixer (M-256, South China University of Technology, Guangzhou, China). Secondly, all liquid lipid sources were mixed and added and rubbed well until no visible lipid particles were present. Finally, 30% distilled water was added, and the feed was extruded into round strips using an expander (G-90A, Beijing Modern Yanggong Machinery Technology Development Co., Ltd., Beijing, China) and cut into small uniform pellets. The prepared feeds were dried in a ventilated place and stored at −20 °C. Finally, the prepared feed pellets were dried in the shaded area and stored in a sealed container at −20 °C before the experiment [17].

### 2.2. Experimental Design

Juvenile groupers were sourced from the Yongsheng Fish Hatchery (Zhanjiang, Guangdong, China). All groupers were placed in many fiberglass tanks (2 m^3^) for two weeks before the start of the experiment to adapt to the new environment and meet the experimental requirements. Feeding was stopped 24 h before the start of the experiment t [14]. Two hundred and forty evenly sized groupers with an initial weight of 9.10 ± 0.03 g were selected and randomly divided into 8 tanks with 30 fish in each tank for 2 groups to perform the 8-week feeding trial. During the experimental period, water was changed daily to keep the water fresh, and fish were fed twice a day (7:30 and 17:30).

At the end of the culture experiment, all groupers were fasted for 24 h. Fifteen fish per tank were selected to start the salinity stress experiment in water with a salinity of 10‰.

### 2.3. Sample Collection

Five groupers were selected from each tank at each sampling time (0, 6, 12, and 24 h). Livers were extracted for antioxidant index and gene expression, and intestines were taken for intestinal flora determination.

### 2.4. Liver Antioxidant Index

The content of malondialdehyde (MDA) and activity of superoxide dismutase (SOD) were determined by commercial ELISA kits (Shanghai Enzyme-linked Biotechnology Co., Ltd., Shanghai, China).

### 2.5. Real-Time Quantitative RT-PCR Analysis of Gene Expression

All mRNA expression in total RNA was extracted from the liver using RNA Extraction Kit (TansGen Biotech, Co., Ltd., Beijing, China), and the RNA concentration was determined by nuclear acid protein analyzer (Guangzhou Yitao Scientific Instrument Corporation, Guangzhou, China). Complementary DNA (cDNA) was synthesized by Evo M-MLV RT kit (Accurate Biotechnology (Hunan) Co., Ltd., Hunan, China). All gene expressions of interleukin-6 (IL6), catalase (CAT) and SOD were performed on Roche Light Cycler 480II (Switzerland) using an SYBR^®^ Green Pro Taq HS Premix II kit (Accurate Biotechnology (Hunan) Co., Ltd., Hunan, China). Primer sequences were designed using Primer 5 following the cDNA sequences found in NCBI (Table 2). The results of real-time qPCR were analyzed by the 2^−ΔΔCT^ method [18].

### 2.6. Intestinal Flora Analysis

#### 2.6.1. Extraction of Genomic DNA

The DNA from intestinal sample was extracted by Guangzhou Gene Denovo Ltd. using the E.Z.N.A fecal DNA Kit (Omega Bio-Tek, Norcross, GA, USA).

#### 2.6.2. PCR Amplification of Target Regions

After extraction of genomic DNA from the samples, the V3 + V4 region of the 16S rDNA was amplified using specific primers with barcodes. The primer sequences were: 341F: CCTACGGGGNGGCWGCAG; 806R: GGACTACHVGGGTATCTAAT. PCR reaction conditions were set according to the description of the previous study.

#### 2.6.3. PCR Product Purification, Library Construction and Sequencing

PCR products were first examined by 2% agarose gel electrophoresis then purified with AxyPrep DNA Gel Extraction Kit (Axygen Biosciences, Co., Ltd., Beijing, China) following the instructions. The purified amplification products were ligated to sequencing ligations, and sequencing libraries were constructed and sequenced on Illumina.

#### 2.6.4. Data Analysis and Functional Prediction

After sequencing the raw reads, the low-quality reads and tags were first filtered using the Usearch software, and the resulting data are called Clean Tag. Secondly, clustering was conducted by applying the Usearch software to access OTU abundance and OTU representative sequences according to Clean Tag. Finally, functional predictions such as species composition analysis, indicator species analysis, and alpha diversity analysis were performed on the basis of OTU sequences and abundance data.

### 2.7. Statistical Analysis

Before the application of ANOVA, the homogeneity of variance was analyzed. Data from this experiment were statistically analyzed using SPSS 17.0 software. One-way ANOVA was used to analyze the data, and if there were significant differences (*p* < 0.05), Duncan’s multiple comparisons were adopted. The experimental data were expressed as mean ± standard deviation (X ± SD).

## 3. Results

### 3.1. Effects of GML on Antioxidant Enzyme Activities Caused by Salinity Changes for Grouper

**Figure 1:**
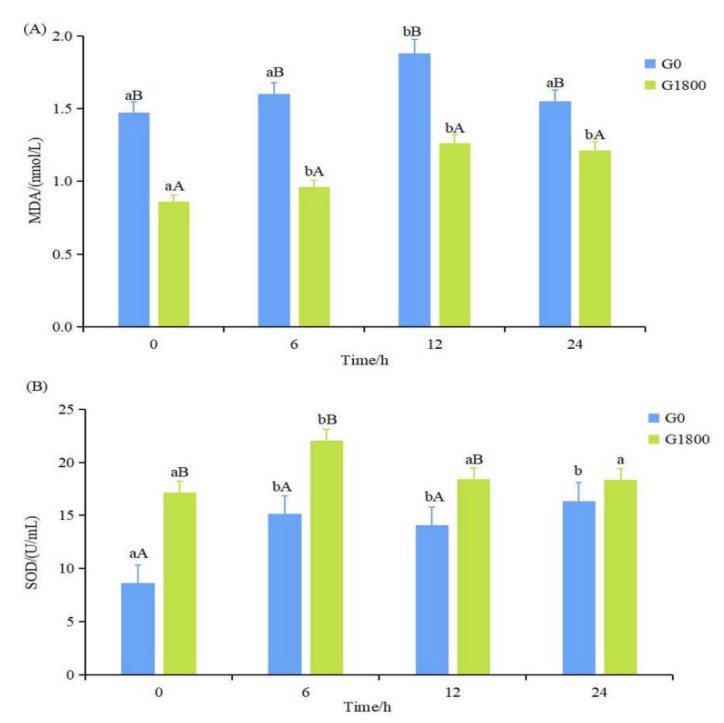
Effect of dietary GML supplementation on MDA content (**A**) and SOD activity (**B**) of grouper under salinity stress. Data represent means of three tanks in each group; error bar indicates S. D. Values with different letters are significantly different (*p* < 0.05). “a, b” represents the significance between the same group at different times; “A, B” represents the significance between different groups at the same time period. The same as below. MDA: malondialdehyde; SOD: superoxide dismutase.

As seen from Figure 1A, the MDA content of the G1800 group was significantly lower than that of the G0 group during the salinity change experiment (*p* < 0.05). The MDA content of the G0 and G1800 groups as a whole increased first and then decreased, and it had the highest value at 12 h. As shown in Figure 1B, the SOD activity in the G1800 group at 0, 6, and 12 h was significantly higher than that in the G0 group (*p* < 0.05).

### 3.2. Effects of GML on Gene Expression Caused by Salinity Changes in Grouper

**Figure 2:**
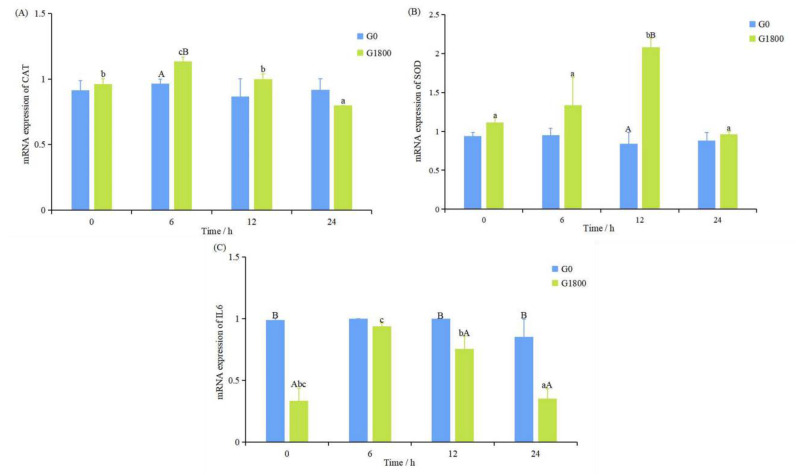
Effect of dietary GML supplementation on the expression of CAT (**A**), SOD (**B**), and IL6 (**C**) in grouper under salinity stress. Data represent means of three tanks in each group; error bar indicates S. D. Values with different letters are significantly different (*p* < 0.05). “a, b, c” represents the significance between the same group at different times; “A, B” represents the significance between different groups at the same time period. CAT: superoxide dismutase; SOD: superoxide dismutase; IL6: interleukin 6.

The expressions of CAT, SOD, and IL6 in the G1800 group all increased and then decreased with time. The expression of CAT reached its highest value at 6 h (*p* < 0.05, Figure 2A) and SOD at 12 h (*p* < 0.05, Figure 2B), both of which were significantly higher than that of the G0 group. The expression of IL6 reached its highest value at 6 h (*p* < 0.05) and was significantly lower than that of the G0 group at 0, 12, and 24 h (*p* < 0.05, Figure 2C).

### 3.3. Effects of GML on Intestinal Flora Caused by Salinity Changes in Grouper

#### 3.3.1. Effects of GML on Relative Abundance at the Phylum Level Caused by Salinity Changes in Grouper

**Figure 3:**
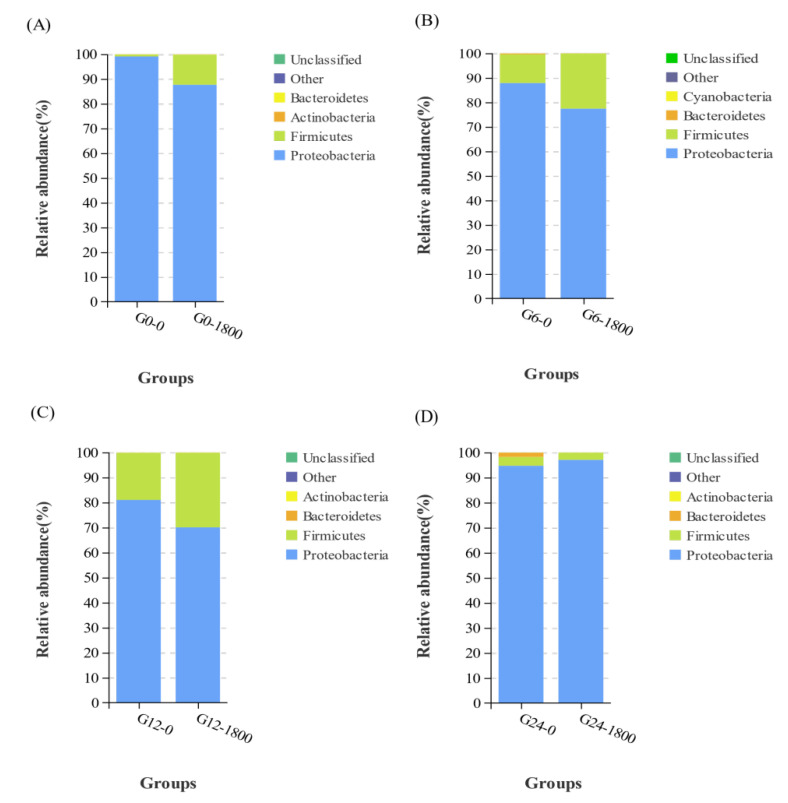
Effect of dietary GML supplementation on the relative abundance of Phylum in grouper intestinal flora under salinity stress. (**A**), (**B**), (**C**), and (**D**) represent the relative abundance of Phylum level of G0 group and G1800 group at 0 h, 6 h, 12 h, and 24 h, respectively.

At 0, 12, and 24 h, the intestinal flora of grouper was mainly composed of Proteobacteria, Firmicutes, Bacteroidetes, and Actinobacteria (Figure 3A,C,D).

By contrast, the intestinal flora composition of grouper changed when the stress time reached 6 h, and the top four flora changed to Proteobacteria, Firmicutes, Bacteroidetes, and Cyanobacteria (Figure 3B). The specific relative abundance values of each group are supplemented in Table 3.

#### 3.3.2. Effects of GML on Relative Abundance at the Genus Level Caused by Salinity Changes in Grouper

The top four genus with the highest relative abundance at 0, 6, and 12 h were *Photobacterium*, *Acinetobacter*, *Bacillus*, and *Vibrio*, but the order was different; while *Acinetobacter* and *Vibrio* were still the top four at 24 h, the relative abundance of *Bacillus* and *Photobacterium* was significantly reduced and replaced by *Proteus* and *Morganella*.

**Figure 4:**
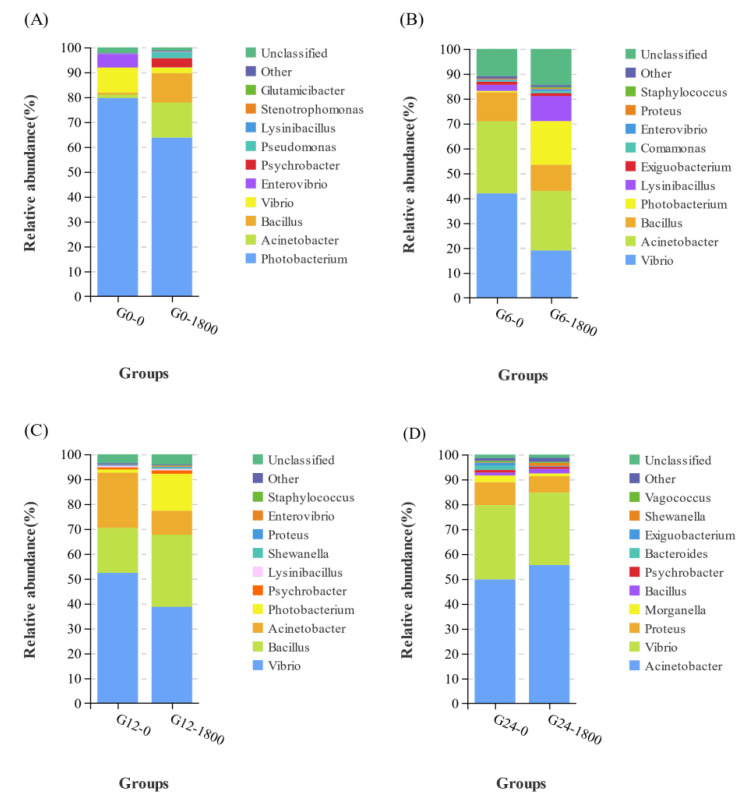
Effect of dietary GML supplementation on the relative abundance of Genus in grouper intestinal flora under salinity stress. (**A**), (**B**), (**C**), and (**D**) represent the relative abundance of Genus level of G0 group and G1800 group at 0 h, 6 h, 12 h, and 24 h, respectively.

At 0 h, the top four groups with relative abundance in each group were *Photobacterium*, *Acinetobacter*, *Bacillus*, and *Vibrio* (Figure 4A). At 6 h, the top four groups with relative abundance in each group were *Vibrio*, *Acinetobacter*, *Bacillus*, and *Photobacterium* (Figure 4B). At 12 h, the top four groups with relative abundance in each group were *Vibrio*, *Bacillus*, *Acinetobacter*, and *Photobacterium* (Figure 4C). At 24 h, the top four groups with relative abundance in each group were *Acinetobacter*, *Vibrio*, *Proteus*, and *Morganella* (Figure 4D).

#### 3.3.3. Effects of GML on Indicator Species Caused by Salinity Changes in Grouper

**Figure 5:**
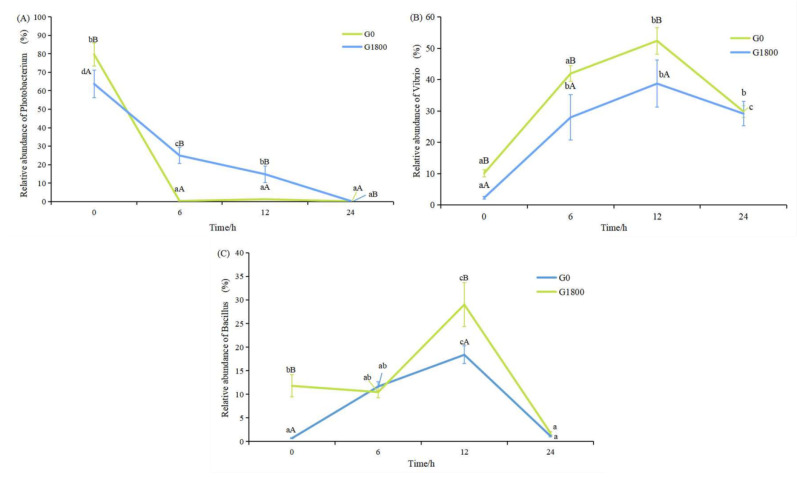
The effect of adding GML to diet on the indicator species of *Photobacterium* (**A**), *Vibrio* (**B**), and *Bacillus* (**C**) in grouper intestinal flora under salinity stress. Data represent means of three tanks in each group; error bar indicates S. D. Values with different letters are significantly different (*p* < 0.05). “a, b, c” represents the significance between the same group at different times; “A, B” represents the significance between different groups at the same time period.

The relative abundance of *Photobacterium* in the G0 and G1800 groups decreased significantly (*p* < 0.05) with increasing time throughout the salinity change period. At 0 h, the abundance in the G0 group was significantly higher than that in the G1800 group. At 6, 12, and 24 h, the abundance in the G1800 group was significantly higher than that in the G0 group (*p* < 0.05, Figure 5A).

The relative abundance of *Vibrio* in the G0 and G1800 groups increased with time and demonstrated the highest value at 12 h (*p* < 0.05). The *Vibrio* in the G1800 group was significantly lower than that in the G0 group, except for 24 h (*p* < 0.05). The relative abundance of *Bacillus* in the G0 group increased with time and exhibited the highest value at 12 h *(p* < 0.05). Meanwhile, the *Bacillus* in the G1800 group showed no significant difference at 0, 6, and 24 h, but it increased significantly at 12 h (*p* < 0.05).

## 4. Discussion

The antioxidant system in aquatic animals consists of an enzymatic system (SOD and CAT) and a non-enzymatic system (oxidation products such as MDA and other non-enzymatic antioxidants). SOD is the first line of defense against oxidative stress, scanning excess oxygen radicals (ROS), and increasing the antioxidant level in fish, which, in turn, protects cells from ROS damage [19]. ROS could react with phospholipids, enzymes, and other macromolecules on biological membranes to form lipid peroxidation products, which expose cells to oxidative damage. MDA is one of the lipid peroxides, and it reflects the degree of oxidative damage in the organism [20]. The addition of 2000 mg/kg GML to the diet of *Eriocheir sinensis* significantly increased the SOD activity and decreased the MDA content in the hepatopancreas [21]. Wang et al. [12] experimentally demonstrated that adding 350 mg/kg GML significantly increased the SOD activity of *Litopenaeus vannamei*. In the present study, compared with the control group, supplementation of 1800 mg/kg GML significantly increased the SOD activity of grouper, improved the antioxidant capacity, and enhanced the ability to remove ROS, while significantly reducing the MDA content, decreasing lipid peroxidation, alleviating oxidative stress, and reducing damage in grouper.

CAT belongs to one of the antioxidant enzymes, which could coordinate with SOD to scavenge ROS and prevent or repair oxidative damage [22]. Wang et al. [12] showed that the addition of 350 mg/kg GML to the diet significantly increased the SOD and CAT activities of *Litopenaeus vannamei*. In the salinity stress experiment, the expression levels of CAT and SOD in the G1800 group increased and then decreased, and they had the highest values at 6 and 12 h, respectively, both of which were significantly higher than those in the G0 group. This finding indicated that the peroxidation level was alleviated by the addition of GML, which regulated the delicate balance between oxidants and antioxidants, enhanced the antioxidant capacity of grouper, and reduced oxidative stress. IL6 is one of a family of pro-inflammatory factors that could significantly promote pathological inflammation [23]. Studies have shown that IL6 expression tends to decrease after adding 750 mg/kg GML to *Danio rerio* diets [9]. Zhao et al. [24] showed that adding 600 mg/kg GML reduced the serum IL6 concentration and decreased the inflammatory response in high-fat-fed mice. The results of the present work show that supplementation of GML significantly reduced the expression of IL6 in the liver of grouper, except for 6 h during the salinity stress experiment, similar to the results of previous studies. Oxidative stress could activate multiple transcription factors, leading to differential expression of genes related to inflammatory pathways. The inflammation caused by oxidative stress is the etiology of many chronic diseases [25,26]. Inflammatory diseases could reduce animal production performance, reproductive performance, and disease resistance. They not only cause huge economic losses to farmers but also endanger the health of consumers [27,28]. The present study demonstrated that dietary addition of GML could alleviate the increase in MDA content caused by sudden change in salinity and remove the accumulated ROS in the body by increasing the expression of SOD and CAT, reduce the systemic inflammatory response, and attenuate the stress response.

The intestinal flora is a complex ecosystem with dynamic diversity. A close relationship exists between the intestinal flora and the immune system, playing an important role in maintaining normal intestinal function and the immune function of the body [29,30]. Diet is one of the main environmental factors that shape the intestinal flora, and the composition of the intestinal flora could be altered in response to changes in diet [30]. The intestinal flora of grouper consisted mainly of Proteobacteria, Firmicutes, Actinobacteria, and Bacteroidetes, similar to the results of previous studies [17]. The present study showed that the Firmicutes in the intestinal flora of grouper significantly increased after GML supplementation. The main metabolite of Firmicutes is butyric acid (BA) [31]. BA is a major source of energy for intestinal epithelial cells and may promote intestinal health by increasing epithelial absorptive cells [32]. The addition of GML can significantly increase the abundance of Firmicutes, improve BA content, and promote intestinal health. The sudden change in salinity had minimal effect on the relative abundance of Proteobacteria, Firmicutes, and Bacteroidetes. Only a minor change occurred at 6 h, when the relative abundance of cyanobacteria increased, replacing the actinomycete bacteria as the fourth. *Photobacterium* species are pathogenic in a wide range of marine animals, including fish, crustaceans, and mollusks [33]. *Photobacterium* is a Gram-negative bacterium that prefers salinity environments of 2–4%, and it could produce large amounts of histamine in high-salinity environments [34]. Long-term ingestion of diets containing high levels of histamine will lead to excessive production and metabolic imbalance of oxygen radicals in carnivorous fish, reduce the activity of liver antioxidant enzymes, and cause liver oxidative stress and apoptosis of normal hepatocytes, thereby resulting in growth inhibition [35,36]. The abundance of *Photobacterium* was significantly reduced by the addition of GML. The sudden drop in salinity caused the death of *Photobacterium* by water uptake rupture in the G0 and G1800 groups, but the death rate in the G1800 group was significantly lower than that in the G0 group. The reason may be that lauric acid (LA), the hydrolysis product of GML, could enter the liver directly through the portal system in free form to participate in organism metabolism. In the absence of apolipoproteins, LA could also enter the mitochondria for oxidation and rapid energy production for the organism [37]. Osmoregulation is an active transport process that requires adenosine triphosphate (ATP), and GML could rapidly supply energy to provide the ATP needed for osmoregulation, thus increasing osmoregulation capacity and slowly decreasing *Photobacterium* abundance. *Vibrio* is a salt-loving bacteria, a common pathogen of aquatic creatures, and known as the “scourge” of marine fish [38]. The reduction in salinity levels is generally recognized as one of the factors that promote the abundance of *Vibrio* [39]. The generation time of pathogenic *Vibrio* could be as low as 20–30 min, thus allowing a rapid response to changing environmental conditions [40]. Oberbeckmann et al. [41] showed that an abrupt drop in salinity and a peak in algae caused an extremely high abundance of *Vibrio* of γ-Proteobacteria at 24%. The relative abundance of *Vibrio vulnificus* was generally negatively correlated with salinity, and a high abundance of it was observed at 5–10‰ salinity, regardless of temperature [42]. The addition of GML to the diet significantly reduced *Vibrio* abundance. The abundance of *Vibrio* in the G0 and G1800 groups increased and then decreased over time after salinity change, but the *Vibrio* abundance in the G1800 group was consistently significantly lower than that in the G10 group. This finding indicated that GML significantly inhibited the multiplication of pathogenic *V. vulnificus* after a sudden drop in salinity and improved the stress resistance of grouper. *Bacillus* is widely used as a probiotic in aquaculture. Many experiments have shown that *Bacillus* could enhance the activity of antioxidant enzymes, increase the expression of immune-related genes and stress-resistant-related genes, and improve the ability of fish to resist pathogenic microorganisms [43,44,45,46]. The results of the present study showed that adding GML significantly increased the abundance of *Bacillus*. After 12 h, the abundance of *Bacillus* in the G1800 group was significantly higher than that in the G0 group. It slowed down the changes in intestinal flora caused by sudden changes in salinity, increased the abundance of beneficial bacteria, and improved the ability of the organism to resist stress response.

## 5. Conclusions

The supplementation of GML in grouper diet significantly reduced the oxidative damage represented by salinity stress, improved antioxidant capacity, slowed down intestinal flora imbalance, promoted the abundance of beneficial bacteria, and enhanced resistance to oxidative stress. The findings of the present study can add value to the current existing knowledge of beneficial effects of GML in aquaculture. We will make more efforts to produce more research results of GML in aquatic animals.

## Figures and Tables

**Table 1:** Ingredients and chemical compositions of experimental diets (% DM basis).

Ingredients	G0	G1800
Brown fish meal	30.00	30.00
Soybean meal	18.00	18.00
Corn gluten meal	9.50	9.50
Peanut meal	10.00	10.00
Chicken powder	6.00	6.00
Wheat flour	18.59	18.59
Fish oil	2.00	2.00
Soybean oil	2.00	2.00
Soybean lecithin	1.00	1.00
Ca(H_2_PO_4_)_2_	1.00	1.00
Premix	1.00	1.00
Choline chloride	0.50	0.50
Vitamin C	0.05	0.05
Microcrystalline cellulose	0.36	0.18
GML	0.00	0.18
Total	100.00	100.00
Nutrient levels		
Crude protein	49.74	49.66
Crude lipid	9.42	9.37

Premix (g/kg premix): vitamin A 675,000 IU, vitamin D_3_ 180,000 IU, vitamin E 6000 mg, vitamin K_3_ 1200 mg, vitamin B_1_ 900 mg, vitamin B_2_ 1350 mg, vitamin B_6_ 1050 mg and vitamin B_12_ 7.5 mg, calcium pantothenate 4500 mg, nicotinic acid 6750 mg, folic acid 375 mg, biotin 15 mg, vitamin C phosphate 42,860 mg, inositol 10,000 mg, FeSO_4_·H_2_O 64,286 mg, ZnSO_4_·H_2_O 26,283 mg, MnSO_4_·H_2_O 19,688 mg, Ca (IO_3_) _2_·H_2_O 128.6 mg, CoCl_2_·6H_2_O 323 mg, CuSO_4_·5H_2_O 2357 mg, and Na_2_SeO_3_ 87.6 mg.

**Table 2:** Primers pair sequences for real-time qPCR.

Genes	Nucleotide Sequence (5′-3)	Genbank Accession No.
β-actin	F: GATCTGGCATCACACCTTCT R: CATCTTCTCCCTGTTGGCTT	AY510710.2
SOD	F: GTTGGAGACCTGGGAAATGTGACTG R: CCATTGAGGGTGAGCATCTTGTCC	AY735008.1
CAT	F:GCTCTATCCGCTCCTCTTCTCCTC R: GTAGTTCCTGACGACGGTGATGTG	KT884509.1
IL6	F: CAATCCCAGCACCTTCCAC R: CCTGACAGCCAGACTTCCTCT	AFE62919.1

SOD: superoxide dismutase; CAT: catalase; IL6: interleukin 6.

**Table 3:** Relative abundance values at the phylum level for all groups.

Itmes	G0-0	G0-1800	G6-0	G6-1800	G12-0	G12-1800	G24-0	G24-1800
Proteobacteria	99.072	87.608	87.832	77.375	80.998	70.044	94.666	97.028
Firmicutes	0.794	12.205	11.683	22.513	18.841	29.836	3.524	2.908
Actinobacteria	0.050	0.133	0.004	0.003	0.011	0.009	0.015	0.011
Bacteroidetes	0.052	0.029	0.403	0.083	0.112	0.086	1.749	0.027
Cyanobacteria	0.011	0.004	0.056	0.000	0.005	0.002	0.010	0.000
Others	0.020	0.019	0.007	0.008	0.000	0.000	0.001	0.000
Unclassified	0.002	0.002	0.016	0.017	0.033	0.022	0.035	0.026

## Data Availability

The data presented in this study are available on request from the corresponding author. The data are not publicly available due to privacy.
